# Schisandrin B regulates lipid metabolism in subcutaneous adipocytes

**DOI:** 10.1038/s41598-017-10385-z

**Published:** 2017-08-31

**Authors:** Hiu Yee Kwan, Jiahui Wu, Tao Su, Xiao-Juan Chao, Hua Yu, Bin Liu, Xiuqiong Fu, Anfernee Kai Wing Tse, Chi Leung Chan, Wang Fun Fong, Zhi-ling Yu

**Affiliations:** 1 School of Chinese Medicine, Hong Kong Baptist University, Hong Kong, China; 2 Guangzhou Institute of Cardiovascular Disease, Guangzhou Key Laboratory of Cardiovascular Disease, and the Second Affiliated Hospital, Guangzhou Medical University, Guangzhou, China; 3 Institute of Chinese Medicine Sciences, State Key Laboratory of Quality Research in Chinese Medicine, University of Macau, Macau, China

## Abstract

Subcutaneous adipocytes in obese subjects have a lower sensitivity to catecholamine-induced lipolysis and a higher sensitivity to insulin anti-lipolytic effects compared to adipocytes in other adipose depots. Therefore, increasing lipolysis in subcutaneous adipocytes coupled with enhanced fatty acid oxidation may be an anti-obesity strategy. Schisandrin B (Sch B) is one of the most abundant active dibenzocyclooctadiene derivatives found in the fruit of *Schisandra chinensis* which is a commonly prescribed Chinese medicinal herb. We found that Sch B reduced glycerolipid contents in 3T3-L1 adipocytes and subcutaneous adipocytes dissected from DIO mice. Sch B also activated hormone sensitive lipase (HSL) and increased lipolysis in these adipocyte in a protein kinase A-dependent manner. Interestingly, Sch B increased fatty acid oxidation gene expressions in these adipocytes, implying an increase in fatty acid oxidation after treatment. In *in vivo* model, we found that Sch B increased HSL phosphorylation, reduced glycerolipid levels and increased fatty acid oxidation gene expressions in the subcutaneous adipocytes in the DIO mice. More importantly, Sch B significantly reduced the subcutaneous adipocyte sizes, subcutaneous adipose tissue mass and body weight of the mice. Our study provides scientific evidence to suggest a potential therapeutic function of Sch B or *Schisandra chinensis* seed containing Sch B in reducing obesity.

## Introduction

The incidence of obesity has been increasing over the past decades^1^. According to World Health Organization, obesity and overweight are linked to more deaths than underweight worldwide. Obesity and its associated metabolic syndrome have become health, social and economic problems. Obesity is characterized as an excess accumulation of adipose tissue in the body^2^; while central obesity with adipose tissues mainly accumulate in the abdominal subcutaneous and visceral depots^3, 4^. These subcutaneous adipose tissues have strong association with insulin resistance^3–5^, and obese people are prone to metabolic complications^3^.

The canonical role of adipocytes is to serve as regulator to maintain energy balance in the body^6^. Triacylglycerol (TG) is stored in cytosolic lipid droplets in adipocytes during times of energy excess, and is mobilized to release fatty acids *via* lipolysis when energy is needed or under hormonal influence^7^. However, obesity is always associated with reduced response to catecholamine-stimulated lipolysis^8^ because the beta-adrenergic receptor-stimulated lipolysis is impaired in obese subjects^8^. Adipocytes from obese subjects have lower levels of adenylyl cyclase activity under hormonal-stimulated condition when compared with adipocytes from non-obese controls^9, 10^. A study showed that upon dibutyryl cAMP stimulation, the maximum lipolytic capacity in adipocytes isolated from obese subjects was less than that in adipocytes isolated from non-obese subjects^8^. This finding further suggests that the adipocytes from obese subjects have impaired lipolysis response to beta-adrenergic stimulation.

Many mouse models showed that increased lipolysis and fatty acid oxidation within adipocytes reduced body weight. The enhanced lipolysis alone is insufficient to promote weight loss but the liberated fatty acids must be oxidized and generate ATP^6^. The increased lipolysis does not elevate serum fatty acid levels but increases fatty acid oxidation within the adipocytes by activating PPARδ and inducing fatty acid oxidative genes expressions in the adiocytes^11^. Therefore, these mice have increased lipolysis, enhanced fatty acid oxidation and a lean phenotype^7, 12–14^. A study showed that adipose-tissue-specific adipocyte triglyceride lipase (ATGL) overexpressing mice were leaner, with smaller adipocyte sizes and decreased TG contents in adipose tissues compared to control mice^15^. Another animal model showed that adipose-specific targeting of the pseudokinase Tribbles 3 resulted in enhanced lipolysis and fatty acid oxidation, which protected the mice from diet-induced obesity^16^. Early example of transgenic mice with enhanced adipocyte fatty acid oxidation also resulted in leanness^17^. All these studies suggest that regulating adipocyte lipid metabolism, increasing lipolysis coupled with enhanced fatty acid oxidation within adipocytes is a promising strategy to reduce obesity.

Schisandrin B (Sch B) is one of the most abundant and active dibenzocyclooctadiene derivatives found in the fruit of *Schisandra chinensis*. *Schisandra chinensis* (Turcz.) Baill, can be found in northern China, Japan, Korea and adjacent areas in Russia, and has been used as a herbal medicine in health care^18^. Up to present, it has been reported that Sch B possesses hepatoprotective property^19, 20^, reduces hepatic lipid content^21^, improves glucose homeostasis and enhances hepatic insulin sensitivity^22^. However, its functional role in regulating adipocyte lipid metabolism has not been studied. In this study, we examined the functional role of Sch B in reducing subcutaneous adipose tissue mass by regulating the adipocyte lipid metabolism.

## Results

### Sch B reduces lipid content in 3T3-L1 adipocytes

**Figure 1:**
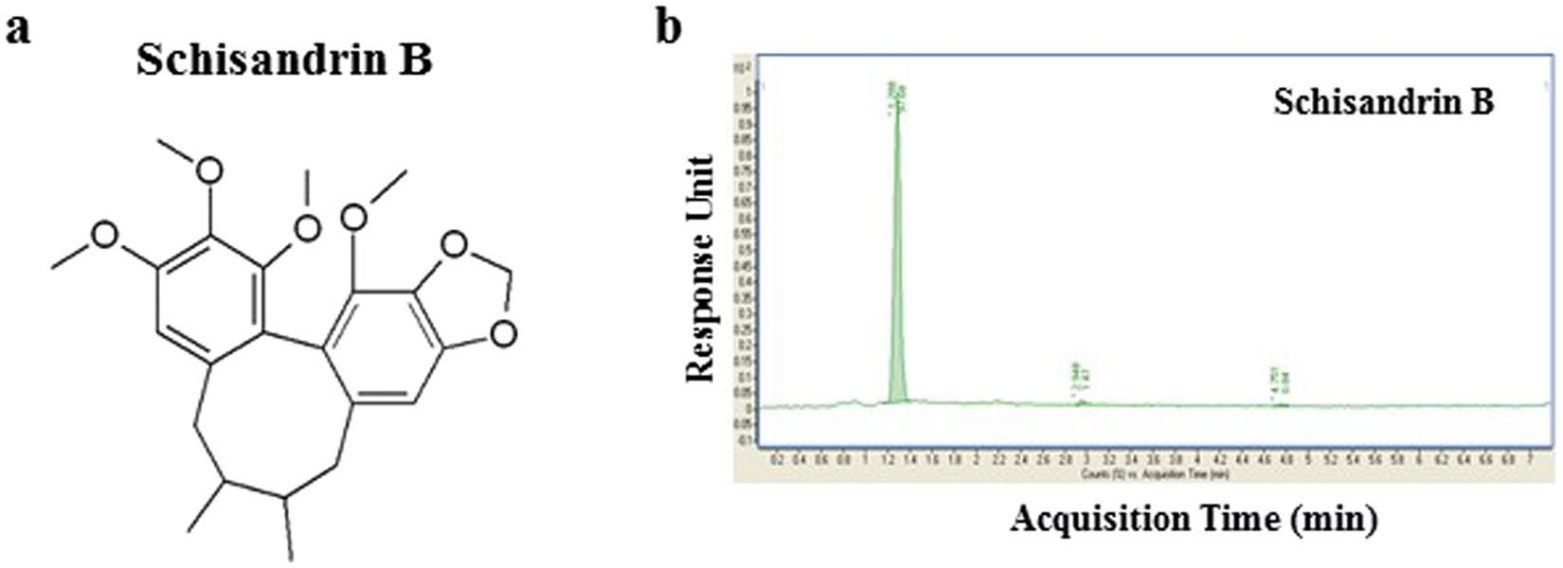
Schisandrin B. (**a**) Schisandrin B (Sch B) structure; (**b**) chromatogram of Sch B in UHPLC analysis.

Sch B is a dibenzocyclooctadiene derivative (Fig. 1a). We first used UHPLC analysis to confirm the purity of Sch B which was 97% (Fig. 1b).

**Figure 2:**
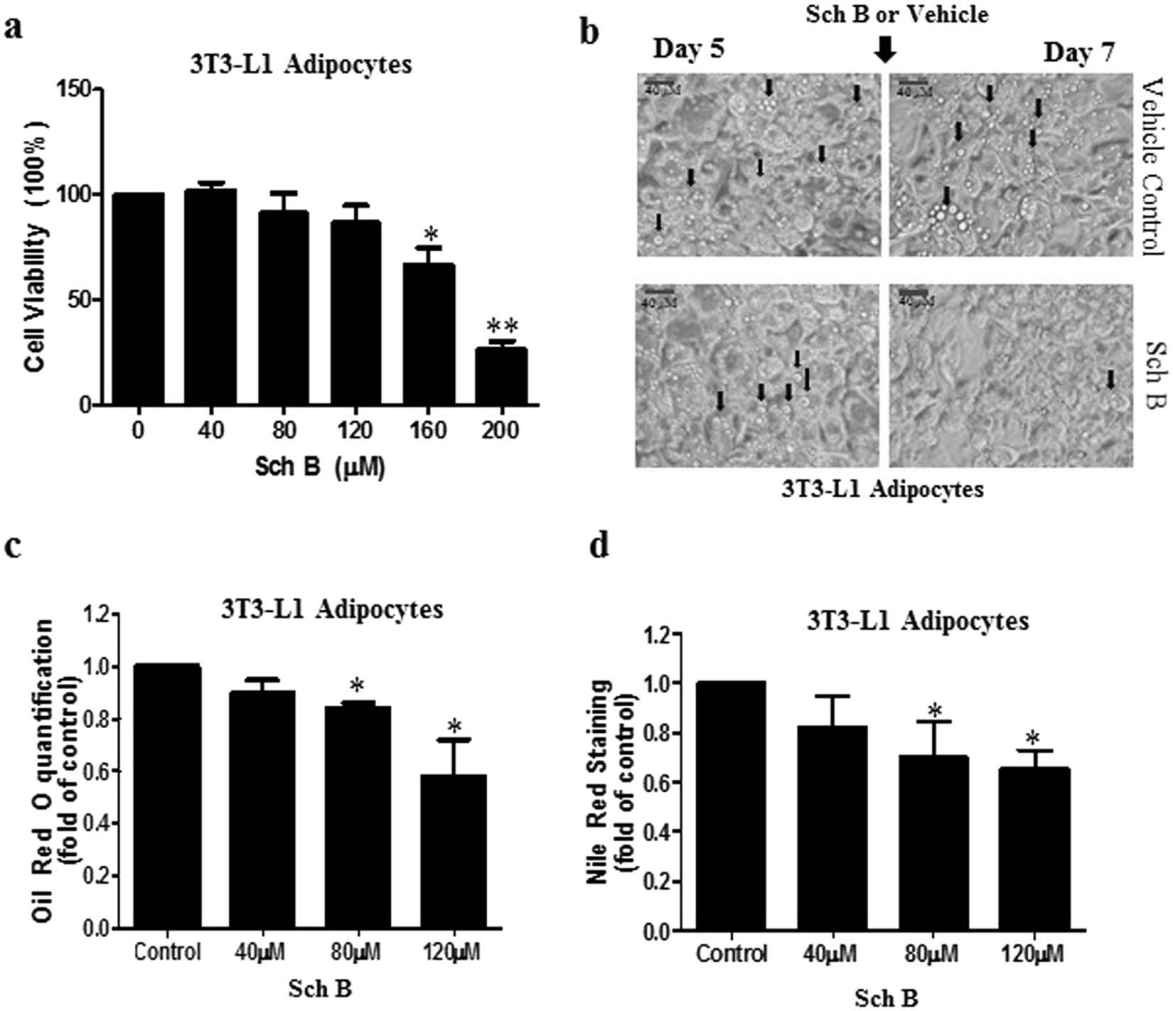
Sch B reduces lipid contents in 3T3-L1 adipocytes. (**a**) Cell viability test of Sch B on 3T3-L1 adipocytes. (**b**) Lipid droplets in the control and Sch B-treated (80 µM) 3T3-L1 adipocytes. Quantification of lipid staining in control and Sch B-treated 3T3-L1 adipocytes by (**c**) Oil Red O and (**d**) Nile Red. Shown is the mean ± SE, n = 3 individual experiments. *p < 0.05, **p < 0.001 compared with control.

In this study, we examined if Sch B regulated lipid metabolism in white adipocytes. We used 3T3-L1 adipocytes as an *in vitro* model. To find out the sub-cytotoxic concentration of Sch B for the experiments, we treated the 3T3-L1 adipocytes with Sch B at different concentrations for 24 hours and performed MTT assay. As shown in Fig. 2a, the IC_50_ of Sch B for 3T3-L1 adipocytes was 172.8 µM. Then, we treated the 3T3-L1 adipocytes with Sch B at 80 µM on the fifth day during the course of differentiation. Interestingly, we found that Sch B reduced the numbers of lipid droplet (Fig. 2b) and the lipid contents in these adipocytes (Fig. 2c,d). These data suggest that Sch B regulates lipid metabolism in 3T3-L1 adipocytes.

### Sch B changes the lipid profiles and reduces glycerolipid levels in 3T3-L1 adipocytes

**Figure 3:**
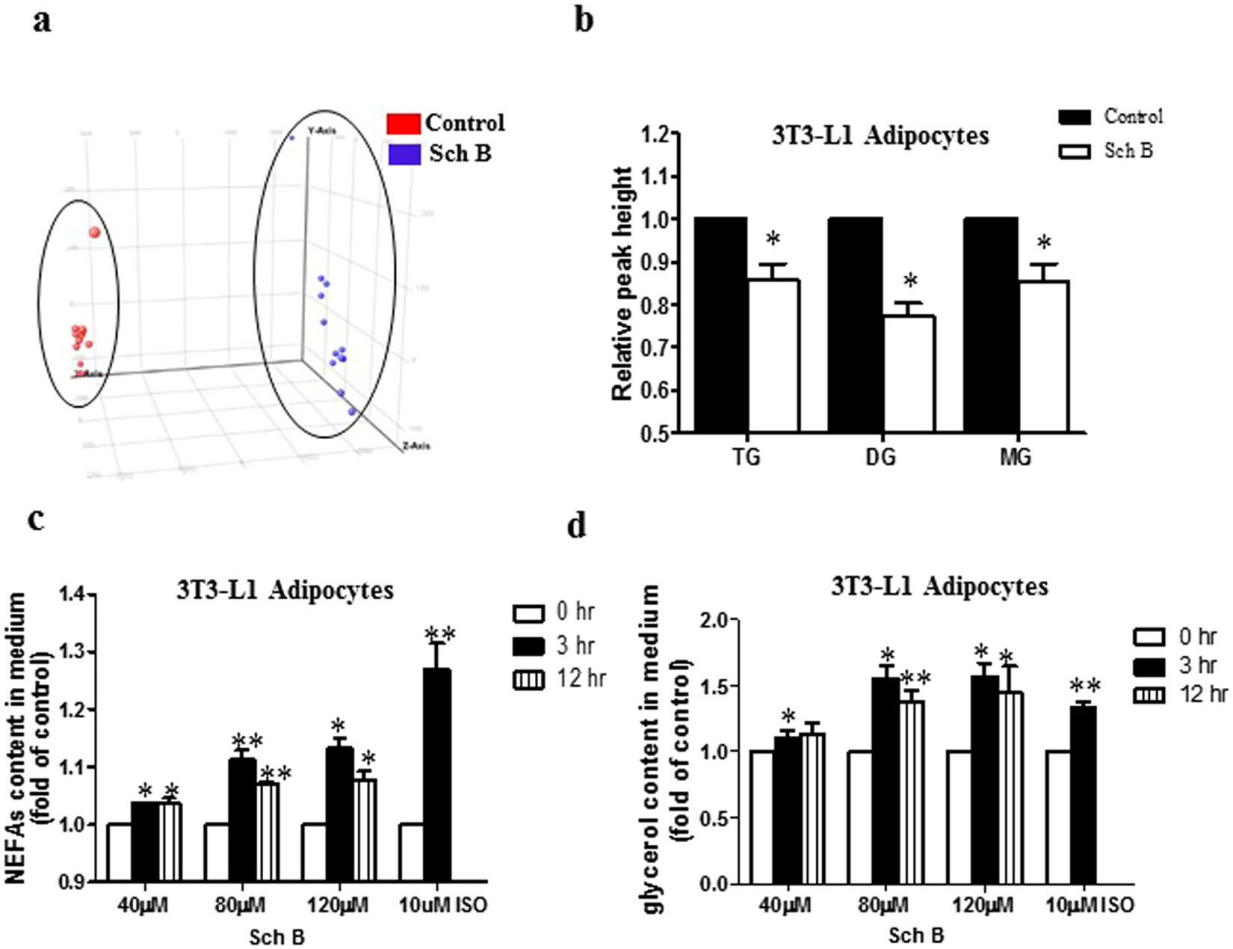
Sch B changes the lipid profile in 3T3-L1 adipocytes. (**a**) Principle component analysis (PCA), and (**b**) triacylglycerol (TG), diacylglycerol (DG) and monoacylglycerol (MG) levels in vehicle and Sch B-treated (80 µM) 3T3-L1 adipocytes. (**c**) Non-esterified fatty acids (NEFAs) and (**d**) glycerol released in 3T3-L1 adipocytes. Isoproterenol (10 µM) was used as positive control. Shown is the mean ± SE, n = 3 individual experiments. *p < 0.05 compared with control.

Next, we performed global LC/MS-based lipidomics to investigate the effects of Sch B on lipid metabolism in adipocytes. We extracted lipids from the Sch B-treated and vehicle 3T3-L1 adipocytes. The applied LC/MS lipidomics platform allowed the identification of lipid metabolites based on retention time and mass-to-charge-ratio (*m* */* *z*). The chromatographic and mass spectrometric parameters are shown in Supplementary Table S1. To examine any Sch B-driven alternation in lipid profile in 3T3-L1 adipocytes, we performed principle component analysis (PCA) based on the filtered data to evaluate sample clustering according to the group variety. Each spot on the PCA represents a sample indicating its particular metabolic pattern. As shown in Fig. 3a, samples from Sch B treatment group and vehicle group were clustered by three principle components (PCs). Each successive principle component (PC) explained the maximum amount of variance possible and was not accounted for by the previous PCs. The PCA suggests that Sch B treatment alters the lipid profile in the 3T3-L1 adipocytes. Furthermore, we found that Sch B significantly reduced the glycerolipid levels in these adipocytes, including triacylglycerol (TG), diacylglycerol (DG) and monoacylglycerol (MG) (Fig. 3b). We also found that Sch B increased the release of non-esterified fatty acids (NEFAs) (Fig. 3c) and glycerol (Fig. 3d) in the 3T3-L1 adipocytes in both time-dependent and dose-dependent manners. Our data clearly demonstrated that Sch B regulated the lipid metabolism and reduced the glycerolipid levels in the 3T3-L1 adipocytes.

### Sch B increases lipolysis in 3T3-L1 adipocytes by activating hormone sensitive lipase

**Figure 4:**
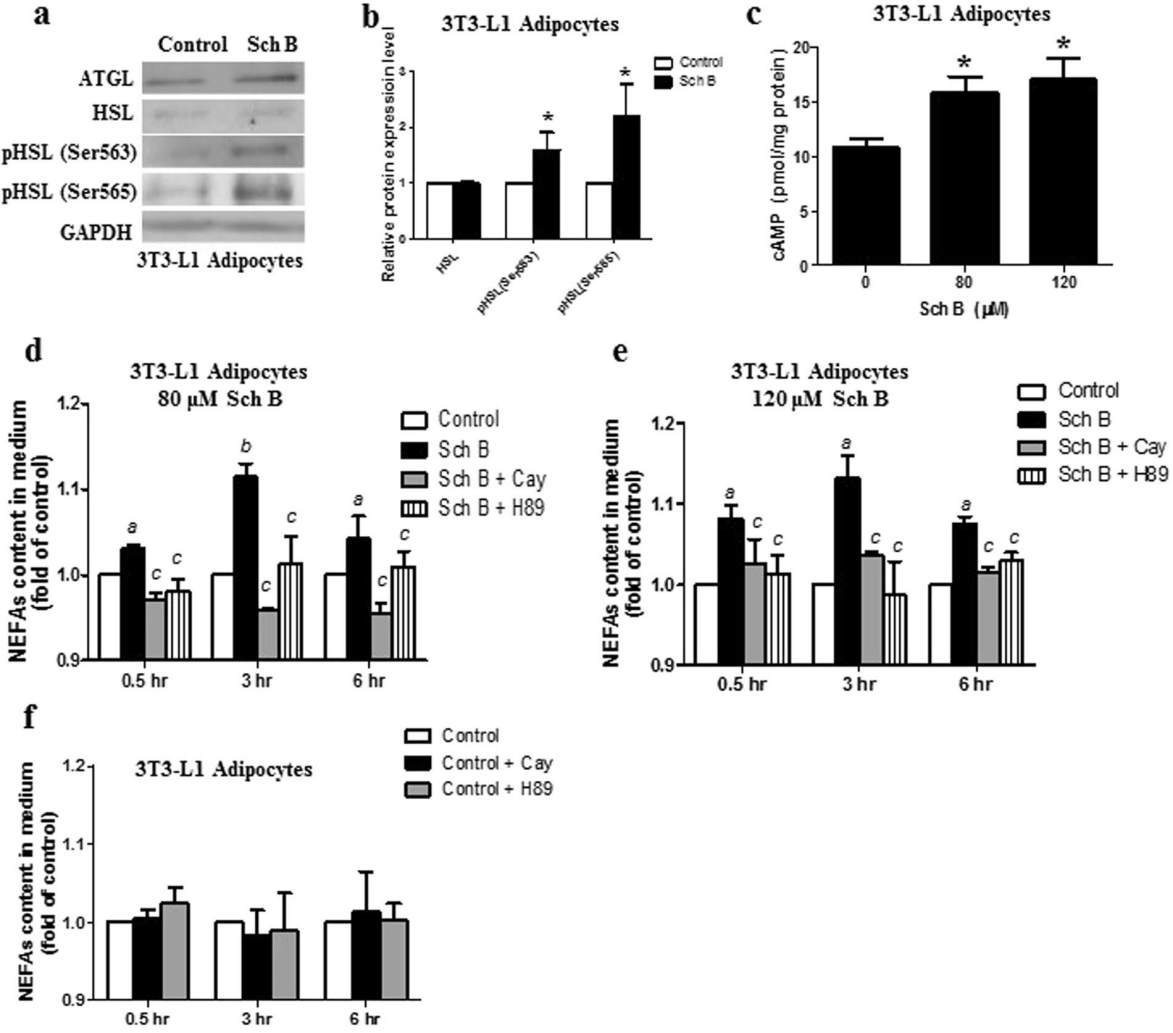
Sch B induces lipolysis in 3T3-L1 adipocytes. (**a**) Representative Western shows the protein expressions of ATGL, HSL and phospho-HSL in control and Sch B-treated (80 µM) 3T3-L1 adipocytes, and (**b**) the quantification of western signal for HSL and p-HSL. (**c**) cAMP levels in control and Sch B-treated 3T3-L1 adipocytes. Shown is the mean ± SE, n = 3 individual experiments. *p < 0.05. Non-esterified fatty acids (NEFAs) released in 3T3-L1 adipocytes after (**d**) 80 µM or (**e**) 120 µM Sch B treatment in the presence or absence of CAY10499 (2 µM) or H89 (10 µM). (**f**) NEFAs released in 3T3-L1 adipocytes in the presence or absence of CAY10499 (2 µM) or H89 (10 µM). Shown is the mean ± SE, n = 3 individual experiments. ^a^ *p* < 0.05 compared to control. ^b^ *p* < 0.001 compared to control. ^c^ *p* < 0.001 compared to Sch B treatment.

Next, we tried to investigate how the glycerolipid levels in 3T3-L1 adipocytes were reduced upon Sch B treatment. Glycerolipids TG can be hydrolyzed to liberate fatty acids and glycerol in lipolysis which is regulated by a step-wise fashion by enzymes ATGL and hormone-sensitive lipase (HSL)^15^. Phosphorylation of HSL on several serine residues activates the enzyme^23^. Therefore, we investigated if Sch B increased lipolysis in 3T3-L1 adipocytes by activating ATGL and HSL. We found that Sch B did not increase the expressions of ATGL and HSL (Fig. 4a and Fig. S1). However, Sch B significantly increased HSL phosphorylation at Ser563 and Ser563 (Fig. 4a,b and Fig. S1). It is reported that phosphorylation of HSL may be mediated by protein kinase A (PKA) activity *via* elevated intracellular cAMP levels^24^. We also found that Sch B elevated the cAMP levels in the 3T3-L1 adipocytes (Fig. 4c), suggesting an involvement of PKA in the Sch B-increased lipolysis. Next, we used Cay10499 a HSL inhibitor, and H89 a PKA inhibitor to examine the involvement of PKA and HSL in Sch B-increased lipolysis. As shown in Fig. 4d,e, inhibition of HSL or PKA significantly reduced Sch B-increased lipolysis in the 3T3-L1 adipocytes; while Cay10499 or H89 alone did not significantly affect the basal NEFAs released in these adipocytes (Fig. 4f). These data suggest that Sch B increases lipolysis in 3T3-L1 adipocytes by activating PKA and HSL.

### Sch B increases fatty acid oxidation gene expressions in 3T3-L1 adipocytes

**Figure 5:**
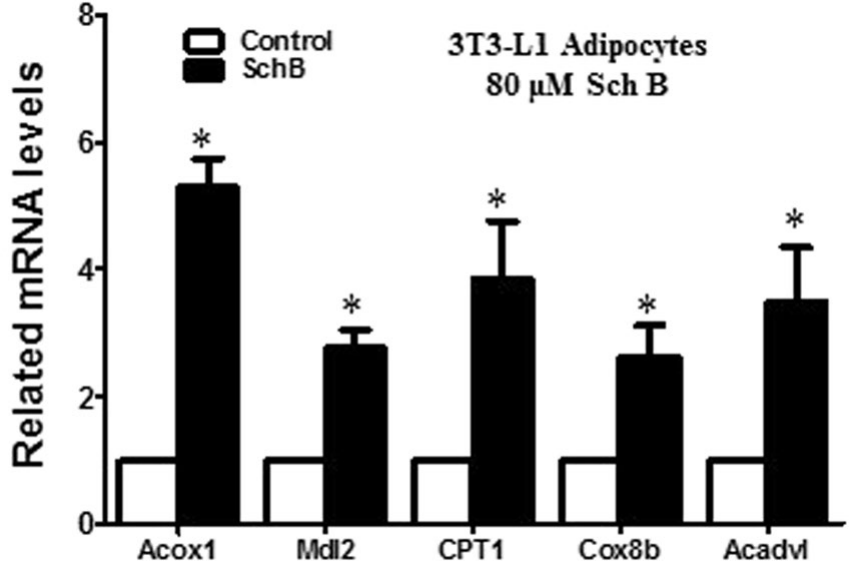
Sch B increases fatty acid oxidation genes expressions in 3T3-L1 adipocytes. (**a**) Expression of acyl-CoA oxidase 1 (Acox1), malate dehydrogenase 2 (Mdl2), carnitine palmitoyltransferase 1 (CPT1), cytochrome c oxidase subunit VIIIb (Cox8b), very-long-chain acyl-CoA dehydrogenase (Acadvl) in control and Sch B-treated (80 µM) 3T3-L1 adipocytes. Shown is the mean ± SE, n = 3 individual experiments. *p < 0.05 compared to control.

Interestingly, we also found that Sch B increased fatty acid oxidation genes expressions in 3T3-L1 adipocytes, including acyl-CoA oxidase I (Acox1), malate dehydrogenase (Mgl2), carnitine palmitoyltransferase (CPT1), cytochrome c oxidase subunit VIIIb (Cox8b) and very-long-chain acyl-CoA dehydrogenase (Acadvl) (Fig. 5). It is reasonable to postulate that Sch B increases fatty acid oxidation in 3T3-L1 adipocytes.

### Sch B activates hormone sensitive lipase in subcutaneous adipocytes dissected from diet-induced obesity mouse model

**Figure 6:**
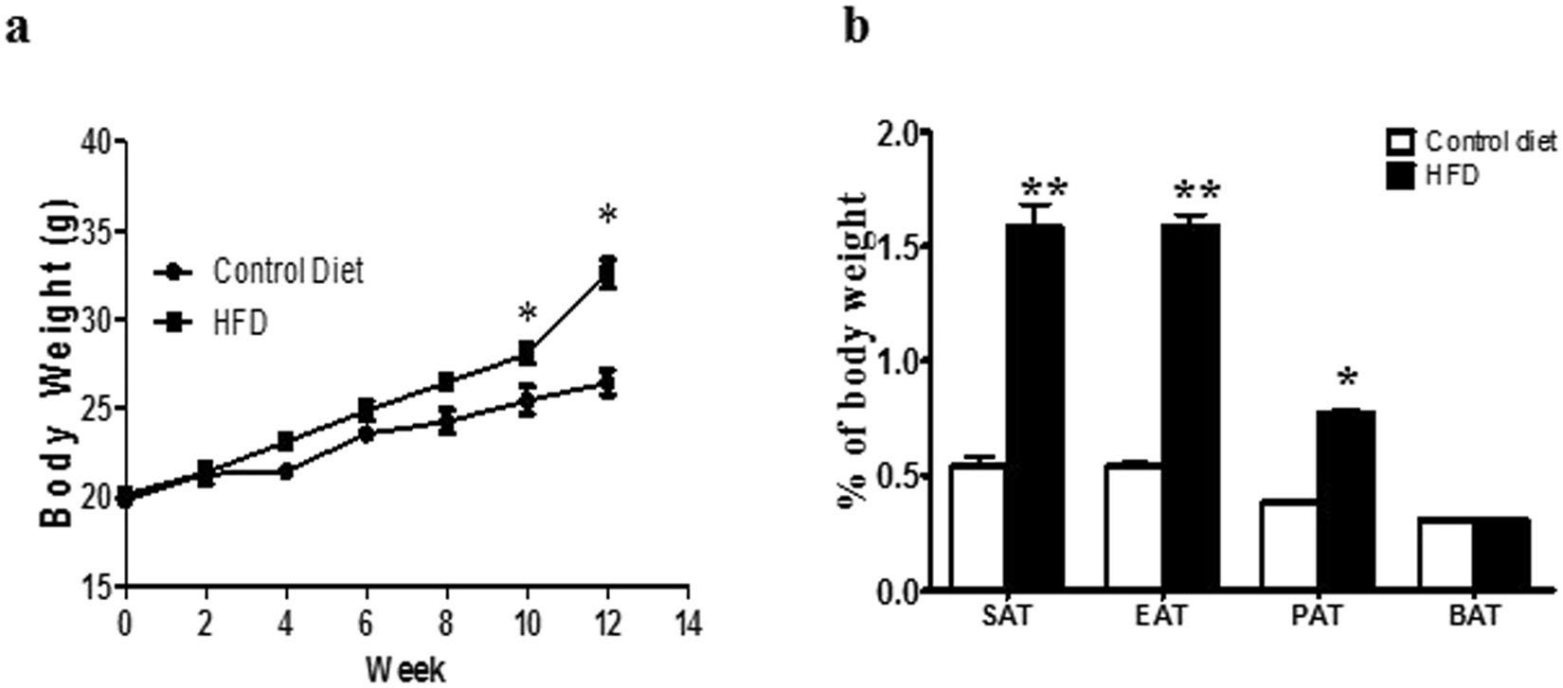
Diet-induced obesity mouse model. (**a**) Body weight and (**b**) different adipose depot weights in the DIO mice. Subcutaneous adipose tissue (SAT), epididymal adipose tissue (EAT), perirenal adipose tissue (PAT) and brown adipose tissue (BAT) in DIO mice. Shown is the mean ± SE, n = 10 mice in each group. *p < 0.05 compared to control.

Our data suggest that Sch B reduces lipid droplets, glycerolipid levels and increases fatty acid oxidation gene expressions in 3T3-L1 adipocytes. Since reduced lipid droplets coupled with increased fatty acid oxidation is an anti-obesity strategy^7, 12–14, 16^, we established a diet-induced obesity (DIO) mouse model for both *ex vivo* and *in vivo* studies to examine the effects of Sch B on adipocyte lipid metabolism. We used DIO mouse model in this study because these DIO mice are not genetically manipulated. Furthermore, they mimics the situation of the nowadays obesity prevalence where most of the individuals develop obesity with excess calories intake. We established the DIO mouse model by feeding C57BL/6 mice high fat diet (HFD) or a matched control diet for 12 weeks. As shown in Fig. 6a, starting from week 10, the body weights of HFD-fed mice were significantly greater than those of the control-diet-fed mice (Fig. 6a). The percentage increase in body weight was 62.06 ± 5.55% for HFD-fed mice, and 33.49 ± 3.41% for control diet-fed mice at week 12. HFD also significantly increased white adipose tissues mass in these mice (Fig. 6b).

**Figure 7:**
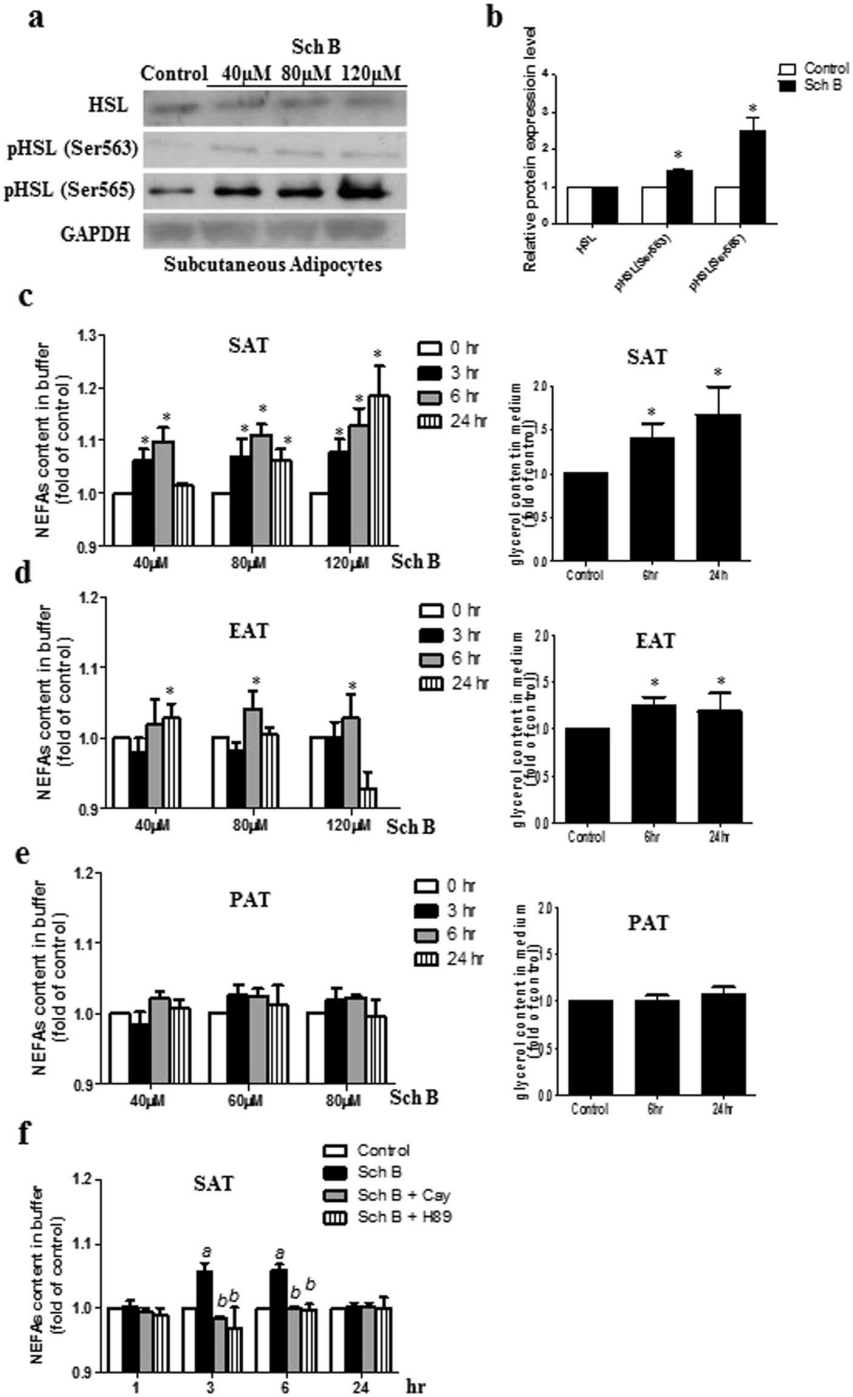
Sch B increases lipolysis in fat pads dissected from DIO mice in *ex vivo* study. (**a**) Representative Western shows the expressions of HSL and p-HSL in subcutaneous adipocytes dissected from DIO mice, and (**b**) quantification of the western signals. NEFAs released in (**c**) subcutaneous adipose tissue (SAT), (**d**) epididymal adipose tissue (EAT) and (**e**) perirenal adipose tissue (PAT) dissected from DIO mice. Shown is the mean ± SE, n = 5 individual experiments. *p < 0.05 compared to control. (**f**) NEFAs released in subcutaneous adipose tissue (SAT) dissected form DIO mice, in the presence or absence of CAY10499 (2 µM) or H89 (10 µM) under Sch B (80 µM) challenge. Shown is the mean ± SE, n = 5 individual experiments. ^a^ *p* < 0.05 compared to control. ^b^ *p* < 0.05 compared to Sch B treatment

First, we performed *ex vivo* study to examine if Sch B regulated adipocyte lipid metabolism in these mice. We isolated the adipocytes from different fat depots dissected from the DIO mice, and then incubated these adipocytes with Sch B at different concentrations for 24 h. Sch B increased HSL phosphorylation at Ser565 and Ser563 (Fig. 7a,b and Fig. S2) in the subcutaneous adipocytes. The treatment also increases NEFAs and glycerol released in the subcutaneous adipose tissue (SAT) (Fig. 7c), which was reduced in the presence of HSL inhibitor or PKA inhibitor (Fig. 7f). Compared with SAT, Sch B had a less significant lipolytic effect in epididymal adipose tissue (EAT) (Fig. 7d) and perirenal adipose tissue (PAT) (Fig. 7e). These data suggest that Sch B activates HSL and increases NEFAs released in subcutaneous adipocytes in *ex vivo* model.

### Sch B regulates lipid metabolism in subcutaneous adipocytes and reduces body weight of the DIO mice

Here, we investigated the effects of Sch B on adipocyte lipid metabolism in *in vivo* model. We treated the DIO mice with Sch B (0.4 g/kg/day) or vehicle for 3 days. Sch B neither affects food intake nor causes apparent adverse effect to the mice.

**Figure 8:**
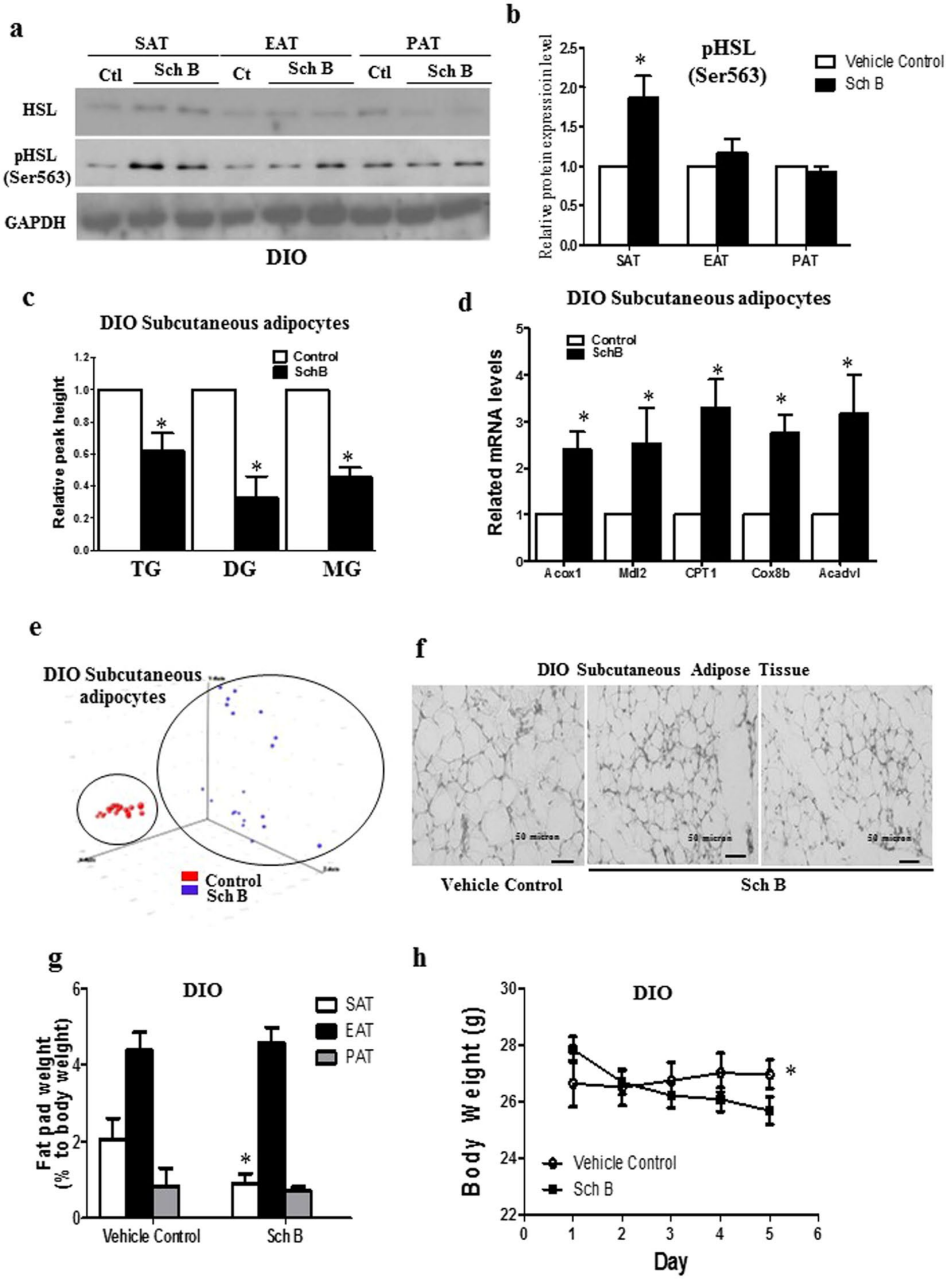
Sch B regulates lipid metabolism in SAT and reduces body weight of the DIO mice. DIO mice were treated with Sch B or vehicle. (**a**) Representative Western shows the expressions of HSL and p-HSL and (**b**) quantification of the Western signals, in the subcutaneous adipose tissue (SAT), epididymal adipose tissue (EAT) and perirenal adipose tissue (PAT) in DIO mice. (**c**) Triacylglycerol (TG), diacylglycerol (DG) and monoacylglycerol (MG) levels in the subcutaneous adipocytes in DIO mice. (**d**) Expressions of acyl-CoA oxidase 1 (Acox1), malate dehydrogenase 2 (Mdl2), carnitine palmitoyltransferase 1 (CPT1), cytochrome c oxidase subunit VIIIb (Cox8b), very-long-chain acyl-CoA dehydrogenase (Acadvl) in the subcutaneous adipocytes in DIO mice. (**e**) Principle component analysis (PCA) of the subcutaneous adipocytes in DIO mice. (**f**) Subcutaneous adipose tissue of the DIO mice. (**g**) Different fat pad weights of the DIO mice. (**h**) Body weight of the DIO mice. Shown is the mean ± SE, n = 10 mice in each group. *p < 0.05 compared to control.

We found that Sch B significantly increased HSL phosphorylation (Fig. 8a,b and Fig. S3), reduced glycerolipid levels (Fig. 8c) and increased fatty acid oxidation gene expressions in the subcutaneous adipocytes (Fig. 8d), suggesting Sch B regulates the lipid metabolism in the subcutaneous adipocytes *in vivo*. Indeed, lipidomics study also showed that Sch B changed the lipid profiles in the subcutaneous adipocytes as revealed by PCA (Fig. 8e). Furthermore, Sch B significantly reduced subcutaneous adipocyte sizes (Fig. 8f), subcutaneous adipose tissue mass (Fig. 8g) and the body weight (Fig. 8h) of the DIO mice. These *in vivo* data suggest that Sch B regulates the lipid metabolism in the subcutaneous adipocytes and reduces the body weight of the DIO mice.

## Discussion

Animal models clearly demonstrate that increased lipolysis coupled with enhanced fatty acid oxidation within adipocytes lead to a lean phenotype^7, 13, 14, 16^. In our study, we showed that Sch B regulated adipocyte lipid metabolism. Sch B increased PKA-mediated HSL activity, increased NEFAs released and fatty acid oxidation gene expressions in subcutaneous adipocytes. The treatment also reduced subcutaneous adipose tissue mass and body weight of the DIO mice. Our data provide scientific evidences to suggest Sch B or *Schisandra chinensis* seed containing Sch B can be a therapeutic agent to reduce obesity.

Subcutaneous adipose tissues have a strong association with metabolic syndrome^3–5^. The accumulation of subcutaneous adipose tissue in obese subjects is partially due to the fact that subcutaneous adipocytes are resistance to lipolysis^3^. HSL controls the regulatory step in lipolysis^11^, HSL expression and activity are lower in the subcutaneous adipocytes than in the adipocytes from other adipose depots^25^. Furthermore, subcutaneous adipocytes have a lower sensitivity to catecholamine-induced lipolysis and a higher sensitivity to insulin anti-lipolytic effects when compared to other adipocytes^3^. Therefore, restricted dietary intake and doing exercise usually reduce visceral adipose tissue much more than subcutaneous adipose tissue^3, 26^. Furthermore, in the obese condition accompanied by insulin resistance, catecholamine-induced lipolysis in white adipocytes is generally disrupted^15, 27^ which also leads to the accumulation of adipose tissues in the obese subjects.

Our study showed that Sch B increased lipolysis and fatty acid oxidation gene expressions in the subcutaneous adipocytes in DIO mice, suggesting a novel therapeutic approach to reduce subcutaneous adipose tissues in obese subjects. Sch B is a natural compound isolated from non-toxic Chinese medicinal herb. Animal studies showed that long-term Sch B treatment had no detectable adverse effect^28^. Here, our data also demonstrated that Sch B treatment neither caused observable adverse effect in mice nor induced apoptosis in adipocytes (data not shown), suggesting Sch B is a safe therapeutic agent.

Our data suggest that Sch B increases PKA-mediated HSL activity in subcutaneous adipocytes. It is known that PKA phosphorylates HSL on multiple sites that causes activation and subsequent translocation of HSL from the cytosol to the lipid droplets^23^. Although HSL is the critical lipolytic enzyme, the PAT family members includes perilipin A, adipose differentiation-related protein (ADFP) and LSDP5 also control lipolysis. These PAT proteins bind HSL through interaction of the lipase with amino acid within the highly conserved amino-terminal PAT-1 domain^29^. ADFP and LSDP5 bind HSL under basal conditions; however, phosphorylation of serine residues within 3 amino-terminal PKA consensus sequence of perilipin A is required for HSL binding and exerts the major control over HSL-mediated lipolysis^29^. Whereas the PKA-mediated phosphorylation on perilipin is affected by A kinase anchoring proteins (AKAP) and AKAP Optical Atrophy 1 (OPA1)^30^. It will be interesting to examine if Sch B affects activities of AKAP and OPA1 and the PKA-mediated phosphorylation on perilipin A in adipocytes. Adipocytes can also secret several factors that regulate lipolysis locally such as TNFα which stimulates lipolysis^7^. Further study can investigate whether Sch B affects TNFα release from adipocytes and hence increases lipolysis. Our data also show that Sch B increases fatty acid oxidation gene expressions in the subcutaneous adipocytes. It has been clearly demonstrated that the fatty acids released from excessive lipolysis activate PPARδ, increase fatty acid oxidation gene expressions in the adipocytes and promote fatty acid oxidation within the adipocytes^11^. Besides, fatty acid oxidation can also be induced by AMPK through phosphorylation and inactivation of acetyl-CoA carboxylase (ACC). Inactivation of ACC alleviates the inhibition on CPT1 mediated by malonyl-CoA, and hence allowing more fatty acids enter into the mitochondria for oxidation. Further study is needed to examine if Sch B affects AMPK and ACC activity in the adipocytes.

Lipolysis coupled with enhanced fatty acid oxidation within adipocytes is an anti-obesity strategy. Delivery of adrenergic active ingredients into the subcutaneous tissue by injecting isoproterenol to stimulate lipolysis in the thigh region in women has been shown to be effective in reducing the thigh regional fat^31^. However, long-term exposure of the adipocytes to beta agonists results in receptor desensitization and down regulation, and eventually reduces lipolytic activity. Furthermore, obese subjects usually develop insulin resistance which further impairs catecholamine-induced lipolysis^15, 27^. Besides, subcutaneous adipocytes themselves are relatively less sensitive to catecholamine-induced lipolysis^3^. Nevertheless, lipolytic technique to reduce regional adiposity by injecting long acting beta-2 adrenergic receptor agonist has been patented (Patent US8420625B2). Partial lipolysis technique by injecting phosphatidylcholine (PC) and deoxycholic acid (DA) mixture is also effective in reducing regional adiposity and clinical studies are on-going in US under US Food and Drug Administration approval^32, 33^. One of the possible underlying mechanisms of actions is the induction of lipolysis by PC^34^. PC is found to increase HSL expression and increase the lipolytic ability of the subcutaneous adipocytes in the obese subjects^34^. The lipolytic products fatty acids may be used by adipocytes, muscle or other tissues for energy production^35^. It is also reported that increased fatty acid oxidation in adipocytes resulted in improved insulin sensitivity^35^. Our study provides a scientific evidence for the further development of Sch B as an anti-obesity therapeutic agent.

The levels of Sch B after Schisandrae administration has been measured in mouse plasma and the data showed that Sch B was maximal in the stomach 15 minutes after intragastric administration and achieved the maximum concentrations in all tissues 2 hours after the administration. With intravenous injection, Sch B was primarily distributed in plasma, liver and kidney 15 minutes after injection. These studies suggest that Sch B is non-toxic in animal models. The distribution of Sch B in subcutaneous adipose tissues after intragastric administration or intravenous injection has not been studied^36^. In our experimental design, we used subcutaneous injection which can help to localize Sch B in the subcutaneous adipose tissues to achieve maximal anti-obesity effect. The dosage of 0.4 g/kg in mice equivalents to a human dose of 0.03 g/kg. Based on the Herbal Medicines Compendium (HMC), published by the U.S. Pharmacopeial Convention (USP), extract of dried ripe fruits of *Schisandra chinensis* (Turcz.) Baill contains not less than 90% and not more than 110% of the total ligands including schisandrin B (γ-schisandrin, C_23_H_28_O_6_), schisandrol B (C_23_H_28_O_7_) and schisandrin A (deoxyschisandrin, C_24_H_32_O_6_) on the dried basis. A study also showed that the lignan contents of the ten samples of *Schinsandra chinensis* collected from different regions in China were different^37^.

Our study clearly demonstrated the Sch B regulated adipocyte lipid metabolism, increased PKA-mediated HSL activity, increased fatty acids released and fatty acid oxidation gene expressions in subcutaneous adipocytes in DIO mice. Our data shed light on the potential therapeutic use of Sch B or *Schisandra chinensis* seed containing Sch B for reducing obesity and hence the obesity-related comorbid conditions.

## Materials and Methods

### Materials

Schisandrin B (Sch B) was purchased from Ningli Technology Co. Ltd (Kunming, China). 3T3-L1 adipocytes were purchased from American Type Culture Collection (ATCC). Dulbecco’s modified essential medium (DMEM), fetal calf serum, penicillin, streptomycin, Hank’s Balanced Salt Solution (HBSS) and Fluo-3/AM were purchased from Life Technologies Limited. Antibodies for adipocyte triglyceride lipase (ATGL), hormone sensitive lipase (HSL), p-HSL (at serine 563 and serine 565), cleaved caspase 3 and GAPDH were purchased from Cell Signaling or Santa Cruz Biotechnology Inc. Dexamethasone, methyisobutylxanthane, insulin, isoproterenol, free glycerol reagent, glucose, fatty acid free BSA, Oil Red O, Nile Red, hematoxylin and eosin, dimethyl sulfoxide and 3-(4,5-dimethylthiazol-2-yl)-2,5-diphenyl tetrazolium bromide (MTT) were purchased from Sigma-Aldrich Chemical Co. H89 was purchased Calbiochem. CAY10499 was purchased from Cayman Chemical. All organic solvents were HPLC grade from Sigma-Aldrich Chemical Co.

### 3T3-L1 cell culture and adipocyte differentiation

3T3-L1 adipocytes were cultured in Dulbecco’s modified essential medium (DMEM) contains 25 mM glucose and supplemented with 10% fetal calf serum and 100 IU/ml penicillin G, and 0.1 mg/ml streptomycin. To induce differentiation, confluent 3T3-L1 cells were treated with differentiation cocktail containing 1 µM dexamethasone, 0.5 mM methyisobutylxanthane and 1.7 µM insulin. After 48 h, the differentiation cocktail was replaced with maintenance medium containing insulin for 2 days before changing to culture medium without insulin^34^.

### Animal handling

All animal experimentation was approved and conducted in accordance with the guidelines from Hong Kong Baptist University and was endorsed by the University Human and Animal Subject Committee and the Department of Health, the Government of Hong Kong Special Administration Region. We purchased male mice C57BL/6 (C57) of 5 weeks old from the Chinese University of Hong Kong. The mice were randomly selected to have either control diet (D12450J Research Diets), or high fat diet (D12762 Research Diets) which was used to induce obesity. Both diet and water were supplied ad libitum. Body weight of each mouse was recorded every week. After 12-week of dietary intervention, the established diet-induced obesity (DIO) mouse models were used for the experiments. For the *in vivo* experiments, DIO mice were given either Sch B by subcutaneous injection of 0.4 g/kg/day in 0.02 ml dimethylsulfoxide or vehicle alone (0.02 ml dimethylsulfoxide) as control for 5 days. Behavioral changes, body weight and food intake of these mice were recorded every day.

### Isolation of adipocytes

Isolation of adipocytes from adipose tissues was performed as described elsewhere^38^. Briefly, adipose tissue dissected from mice were digested for 1 h at 37 °C with collagenase in Krebs-Ringer Buffer (KRB; 12 mM HEPES, 121 mM NaCl, 4.9 mM KCl, 1.2 mM MgSO_4_ and 0.33 mM CaCl_2_) supplemented with 3 mM glucose and 1% fatty acid free BSA, filtered through nylon mesh. Adipocytes were collected from the upper phase after centrifugation. The isolated adipocytes were counted using a haemocytometer^39^.

### Lipolysis

Fat pads from DIO mice were excised and cut into 50 mg samples and incubated at 37 °C without shaking in KRB (12 mM HEPES, 121 mM NaCl, 4.9 mM KCl, 1.2 mM MgSO_4_, 0.33 mM CaCl_2_) containing 2% fatty acid free BSA and 0.1% glucose^38^ in the presence of Sch B or vehicle. At the indicated time point, NEFAs and glycerol release were measured in aliquots from incubation buffer using LabAssay NEFA kit (Wako Chemicals) and free glycerol reagent, respectively^38^. For measuring lipolysis in 3T3-L1 adipocytes, the cells were rinsed with KRB and incubated with KRB supplemented with 2% fatty acid free BSA and 0.1% glucose in the presence of Sch B or vehicle. Isoproterenol was used as positive control. At the indicated time point, NEFAs and glycerol release were measured in aliquots from incubation buffer using LabAssay NEFA kit and free glycerol reagent, respectively. Each treatment was performed in triplicate.

### Cell viability assay

Cytotoxicity of Sch B to 3T3-L1 adipocytes was assessed by 3-(4,5-dimethylthiazol-2-yl)-2,5-diphenyl tetrazolium bromide (MTT) assay. Cells in 96-well plates were treated with Sch B, and 20 μl of MTT solution (5 mg/ml) was added to each well after incubation. The plates were further incubated at 37 °C for 4 h before 100 μl DMSO was added. Optical absorbance was determined at 570 nm with a microplate spectrophotometer. Each treatment was performed in triplicate.

### Oil Red O staining

3T3-L1 adipocytes, with vehicle or Sch B treatment, were stained with freshly prepared Oil Red O working solution for 20 min at room temperature. To quantify staining, Oil Red-O was extracted from the cells with isopropanol containing 4% Nonidet P-40, and optical density was then measured at a wavelength of 520 nm^40^. Each treatment was performed in triplicate.

### Nile red staining

3T3-L1 adipocytes, with vehicle or Sch B treatment, were stained with 1 µM Nile red in Hank’s Balanced Salt Solution (HBSS) for 15 min. Samples of 10000 Nile Red stained cells were counted using a flow cytometer (BD Bioscience)^41^. Each treatment was performed in triplicate.

### Hematoxylin and eosin staining for subcutaneous adipose tissue

SAT were fixed in 10% buffered formalin, embedded in paraffin, cut into 6-µm-thick sections, and stained with hematoxylin and eosin^35^.

### Western blotting analysis

Western blotting analysis was performed as described^40^. Briefly, the nitrocellulose membrane carrying transferred proteins was incubated at 4 °C overnight with corresponding antibody at 1:1000 ratio. Immunodetection was accomplished using horseradish peroxidase-conjugated secondary antibody, followed by ECL detection system (Amersham).

### Real-time polymerase chain reaction analysis

Total RNA was extracted with Trizol reagent (Invitrogen) and treated with DNAase 1 (Invitrogen). RNA (2 μg) was reverse transcribed with oligo-dT using M-MLV reverse transcriptase (Promeg) according to manufacturer’s protocol. Real-time PCR was performed using SYBR green reaction mixture in the ABI 7500 fast real-time PCR system (Applied Biosystemsm). The gene expression data was normalized to the endogenous control β-actin. The relative expression levels of genes were measured according to the formula 2-ΔCt, where ΔCt is the difference in threshold cycle values between the targets and *β*-actin. All samples were analyzed in triplicate.

### cAMP determination

The cAMP levels in adipocytes were measured as described^42^. Briefly, 3T3-L1 adipocytes were treated with either Sch B at the indicated concentration or vehicle as control for 6 h. Adipocytes were then washed with PBS and lysed for cAMP measurement with immunoassay kit (BioVision) following manufacturer’s instructions. The protein quantities of the adipocytes in each group were measured by Bradford method. Each treatment was performed in triplicate.

### Sample preparation for LC/MS

3T3-L1 adipocytes were treated with 120 µM Sch B or DMSO as vehicle for 24 h. Lipids were extracted from these cells for the lipidomics study. To each sample, we added 0.3 ml 0.5 M KH_2_PO_4_, 1.5 ml chloroform and 0.5 ml methanol. After vortex for 2 minutes and centrifugation at 2000 × g, the lower phase was collected and evaporated under a nitrogen stream. The residue was reconstituted in 100 μL of isopropanol-acetonitrile (1:9, v/v) for LC/MS analysis^43^.

### LC/MS analysis and data processing

An Agilent 6540 UHD Accurate-Mass Q-TOF LC/MS mass spectrometer (Agilent Technologies, USA) was connected to an Agilent 1290 Infinity UHPLC *via* an ESI ion source for the analysis of total lipids. An Agilent 6450 Triple Quadrupole LC/MS system accompanied with MassHunter Workstation software (Version B.04.00 Qualitative Analysis, Agilent Technologies) was connected to an Agilent 1290 Infinity UHPLC for specific quantification of targeted bioactive lipids and lipid metabolites^43^. Briefly, we set up a gradient mobile phase comprising solvent TL-A (40% ACN with 10 mM ammonium acetate) and solvent TL-B (acetonitrile: isopropanol, 1:9) with 10 mM ammonium acetate. The raw data were first processed by MassHunter Workstation software (Version B.04.00 Qualitative Analysis, Agilent Technologies). The chromatographic and mass spectrometric parameters for the LC/MS lipidomics study were shown in Table 1. Ions were extracted by molecular features characterized by retention time (RT), intensity in apex of chromatographic peak, and accurate mass. These results were then analyzed by Mass Profiler Professional (MPP) software (Version 2.2, Agilent Technologies). We also set up a filtration platform to further filter the initial entities before doing Principle Component Analysis (PCA). Only entities with abundances above 3000 cps were selected. These entities were then passed a tolerance window of 0.15 min and 2 mDa chosen for alignment of RT and m/z values, respectively. We employed one-way ANOVA to do the statistical analysis. The *p*-value of ANOVA was set to be 0.05 (corresponding with the significance level of 95%)^43^.

## Electronic supplementary material

Supplementary information
