# Supplementary material for: Schisandrin B regulates lipid metabolism in subcutaneous adipocytes
Source: Sci Rep. 2017 Aug 31;7:10266. doi: 10.1038/s41598-017-10385-z (PMC5579161; doi:10.1038/s41598-017-10385-z)
Supplement: Supplementary file 1 — Supplementary information [file 41598_2017_10385_MOESM1_ESM.pdf]

Schisandrin B regulates lipid metabolism in subcutaneous adipocytes

Hiu Yee Kwan[1]*, Jiahui Wu[1], Tao Su[1], Xiao-Juan Chao[1], Hua Yu[1,2], Bin Liu[3], Xiuqiong Fu[1], Anfernee Kai Wing Tse[1], Chi Leung Chan[1], Wang Fun Fong[1], Zhi-ling Yu[1]*

[1]School of Chinese Medicine, Hong Kong Baptist University, Hong Kong, China.

[2]Current address: Institute of Chinese Medicine Sciences, State Key Laboratory of Quality Research in Chinese Medicine, University of Macau, Macau, China.

[3]Guangzhou Institute of Cardiovascular Disease, Guangzhou Key Laboratory of Cardiovascular Disease, and the Second Affiliated Hospital, Guangzhou Medical University, Guangzhou, China.

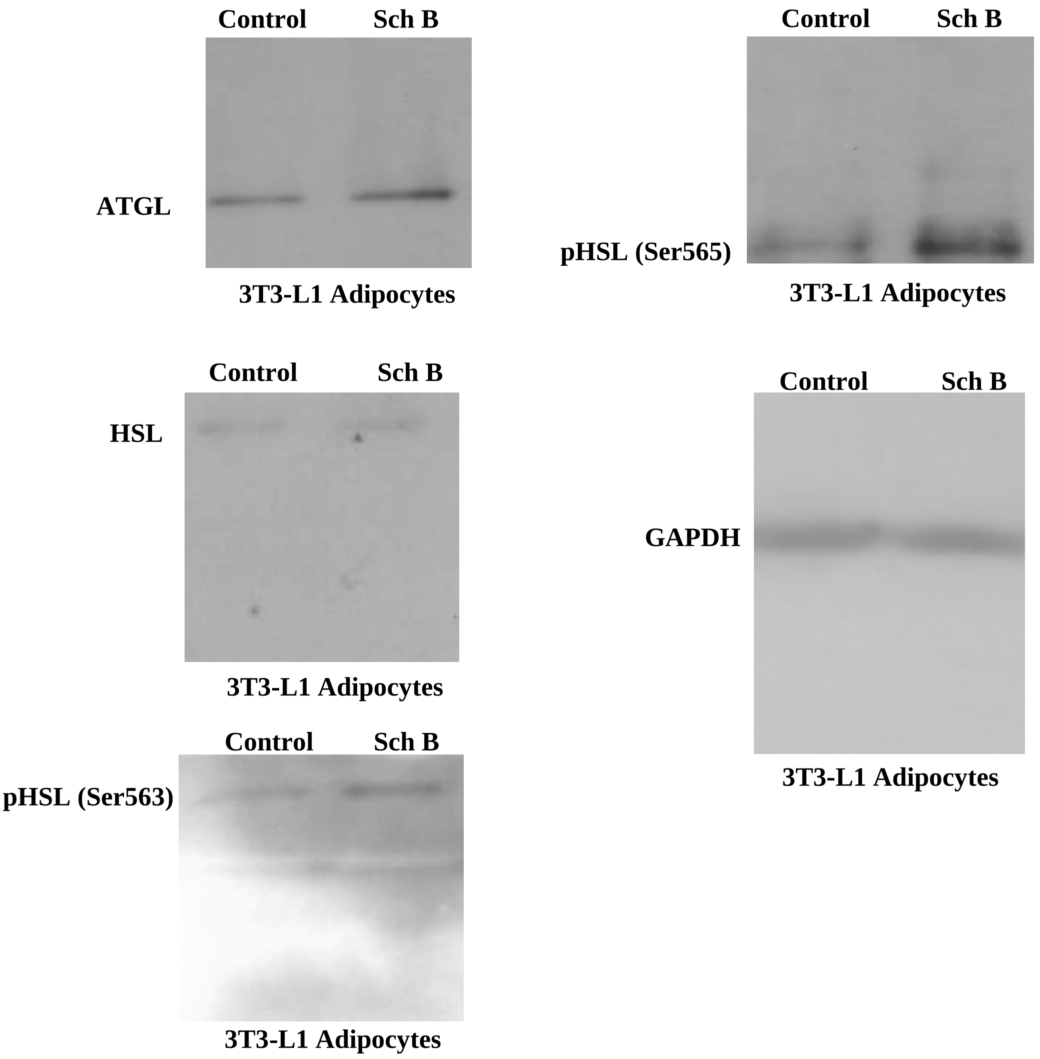

Figure S1. Sch B induces lipolysis in 3T3-L1 adipocytes. Original Western shows the protein expressions of ATGL, HSL and p-HSL in control and Sch B-treated (80µ) 3T3-L1 adipocytes,

Supplementary Figure 1

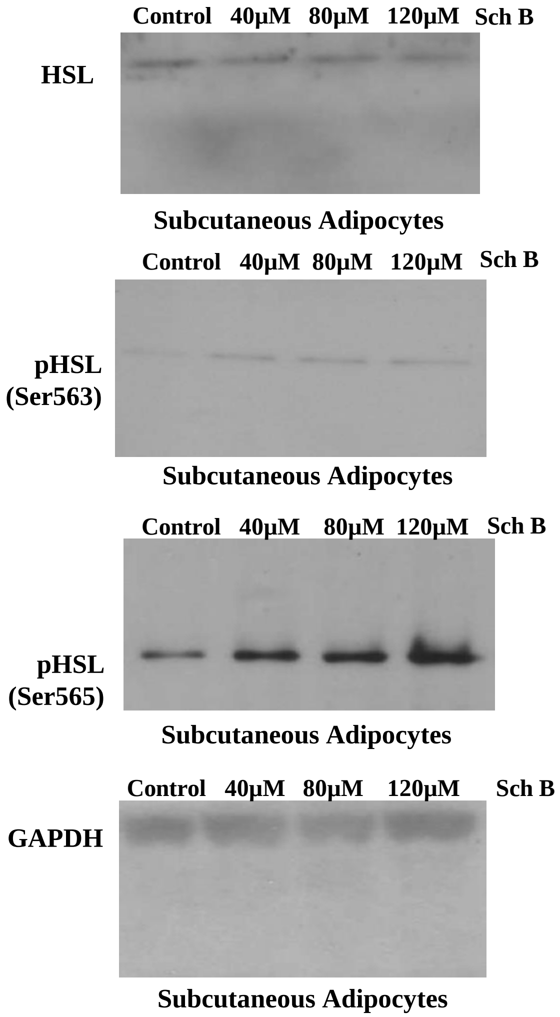

Figure S2. Sch B increases lipolysis in fat pads dissected from DIO mice. Original Western shows the expressions of HSL and p-HSL in DIO mice.

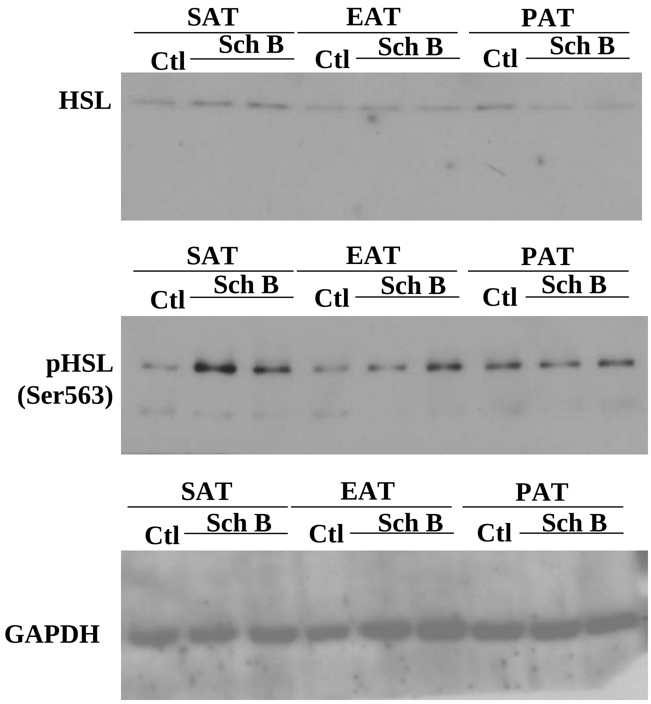

Figure S3 Original Western shows the expressions of HSL and p-HSL and in subcutaneous adipocytes (SAT), epididymal adipocytes (EAT) and perirenal adipose tissue (PAT) in DIO mice after receiving Sch B or vehicle treatment.

Supplementary Figure 3

Table S1: The chromatographic and mass spectrometric parameters

Column	Agilent Eclipse plus
	C_{18} RRHD(2.1mmx50mm,
Mobile phase	1.8µm)
	A: ACN/H_2O (4 : 6) with
	10mM ammonia formate
	B: ACN/ isopropanol (1: 9)
Gradient	with 10mM ammonia formate
	15-25%B (0-5 min),
	25-40%B (5-7 min),
	40-80%B (7-7.5 min),
	80-85%B (7.5-24 min)
Flow rate	85-100%B (24-26min)
Injection volume	400 µL
**ESI parameters**	5 µL
Polarity	
Capillary voltage	Positive
Sheath gas flow rate	4.0 kV
Sheath gas temp.	10 L/min
Drying gas flow	400 °C
rate	6 L/min
Drying gas temp.	300 °C
Nebulizer gas	40 psig
Fragmentor voltage	150 V
Nozzle voltage	300V
Scanning range	150-2000 m/z

Abbreviation: electrospray ionization (ESI)
